# Safety Assessment of *Lactiplantibacillus plantarum* TWK10 Based on Whole-Genome Sequencing, Phenotypic, and Oral Toxicity Analysis

**DOI:** 10.3390/microorganisms10040784

**Published:** 2022-04-07

**Authors:** Han-Yin Hsu, Yi-Chu Liao, Shih-Hsuan Lin, Jin-Seng Lin, Chia-Chia Lee, Koichi Watanabe

**Affiliations:** 1 Culture Collection & Research Institute, SYNBIO TECH INC., Kaohsiung 82151, Taiwan; hanin@synbiotech.com.tw (H.-Y.H.); yc.liao@synbiotech.com.tw (Y.-C.L.); shlin@synbiotech.com.tw (S.-H.L.); jslin@synbiotech.com.tw (J.-S.L.); 2 Department of Animal Science and Technology, National Taiwan University, Taipei 10673, Taiwan

**Keywords:** probiotics, *Lactiplantibacillus plantarum* TWK10, *Lactobacillus plantarum* TWK10, safety assessment, whole-genome sequencing, genotoxicity, oral toxicity

## Abstract

*Lactiplantibacillus plantarum* TWK10 (TWK10), isolated from Taiwanese pickled cabbage, has been demonstrated to exert beneficial probiotic effects in both mice and humans. Here, we comprehensively assessed the safety of TWK10 using both in vivo and in vitro approaches, including whole-genome sequence analysis, an assessment of hemolytic activity, and performing an antimicrobial susceptibility test, the Ames bacterial reverse mutation assay, the chromosomal aberration test, a rodent peripheral blood micronucleus test, and the 28-day subacute oral toxicity assay. The results showed that there was no significant increase in the incidence of reverse mutations or chromosomal aberrations following exposure to TWK10. Moreover, no significant changes were detected either in the number of reticulocytes or the incidence of micronuclei in ICR mice, and no subacute toxicity was recorded in SD rats at the oral TWK10 dosage of 2000 mg/kg body weight/day repeated for 28 days. Additionally, TWK10 exhibited no hemolytic activity and was susceptible to all the antibiotics tested, except kanamycin. However, no antimicrobial resistance genes, virulence factors, or genes involved in biogenic amine synthesis were found in the genome of TWK10. Our findings demonstrated that TWK10 has high potential of being safe for human consumption as a probiotic.

## 1. Introduction

The genus “*Lactobacillus*” previously comprised more than 280 validly described species (https://lpsn.dsmz.de/genus/lactobacillus: accessed on 7 February 2022), representing the largest genus within the community in the members of lactic acid bacteria. Members of this genus are commonly isolated not only from environments associated with fermented food, such as fruit, meat, sourdough, and vegetables, but also the gastrointestinal and vaginal tracts of humans and animals [1,2,3,4]. However, thanks to evidence garnered from phylogenetic analysis based on 16S rRNA gene sequences, it has long been pointed out that the genus “*Lactobacillus*” comprises an extremely heterogeneous population [3]; accordingly, this genus has been reclassified into 25 genera [5], with “*Lactobacillus plantarum*” being assigned into the new genus *Lactiplantibacillus* and designated as *Lactiplantibacillus plantarum*. Strains belonging to this species are widely used in fermented dairy products and food supplements and also as probiotics to enhance human or animal health.

Roy Fuller [6] first defined probiotics as “a live microbial feed supplement which beneficially affects the host animal by improving its intestinal microbial balance”. After repeated modifications, probiotics are now formally defined as “live microorganisms that, when administered in adequate amounts, confer a health benefit on the host” [7,8]. The species *Lactiplantibacillus plantarum (L. plantarum)* has obtained “qualified presumption of safety (QPS)” status from the European Food Safety Authorities (EFSA) [9] and several strains of *L. plantarum* have been granted the “generally recognized as safe (GRAS)” status by the United States Food and Drug Administration (https://www.cfsanappsexternal.fda.gov/scripts/fdcc/?set=GRASNotices: accessed on 7 February 2022).

Over recent years, probiotics have received widespread attention owing to their ability to confer multiple health benefits. The potential benefits of *L. plantarum* as probiotics include exerting immunomodulatory, antioxidant [10], cholesterol-lowering [11], and anti-hypertensive [12] effects, enhancing epithelial defense functions, and maintaining gut microbial homeostasis [13]. However, some lactobacilli also cause human diseases, and the use of probiotics by patients with ulcerative colitis, urinary tract infections, or cardiac valve disease, as well as by immunocompromised patients, may result in infection [14,15]. In addition, characteristics such as adhesion, antimicrobial resistance, and functional properties are strain-specific [16,17,18]. Consequently, because probiotic products are usually designed for daily consumption, the safety of probiotic strains must be strictly evaluated, and possible adverse interactions with the host must be assessed to ensure the safety of probiotic food.

We have previously demonstrated that the administration of *Lactiplantibacillus plantarum* TWK10 (TWK10) exerted beneficial effects on improving exercise performance, increasing muscle mass and strength in both mice and humans, and attenuating age-associated cognitive decline and loss of bone mass in mice. We further found that long-term TWK10 administration did not result in significant histological changes in the organs of mice or alterations in hematological and biochemical parameters in humans [19,20,21,22]. In this study, we comprehensively assessed the safety of TWK10 as a probiotic for human consumption using both in vitro and in vivo approaches. These included undertaking a whole-genome sequence analysis, assessing hemolytic activity, and performing an antimicrobial susceptibility test, the Ames bacterial reverse mutation assay, the chromosomal aberration test, a rodent peripheral blood micronucleus test, and a 28-day subacute oral toxicity analysis.

## 2. Materials and Methods

### 2.1. Bacterial Test Material

TWK10 was isolated from “Pao-tsai”, a traditional Taiwanese pickled cabbage, and identified based on its 16S rRNA gene sequence [23]. Briefly, 25 g of pickled white cabbage was collected aseptically in a sterile plastic bag containing with 225 mL of 0.85% (*w*/*v*) sterile saline and homogenized in a lab-blender (Stomacher 400, Seward, London, UK) for 1 min. Serial 10-fold dilutions were performed, and 0.1-mL aliquots of the appropriate dilutions were directly spread on acidic (pH 5.4) de Man, Rogosa and Sharpe (MRS; BD Difco, Franklin Lakes, NJ, USA) agar plates, and incubated anaerobically at 37 °C for 48 h. Colonies with distinct morphologies (e.g., in terms of color, shape, and size) were selected and purified by streaking at least three times on MRS agar (pH 6.8). A total of 49 isolates were identified based on the 16S rRNA gene sequences, and preserved in MRS broth supplemented with 20% (*v*/*v*) glycerol, at −80 °C, until use. Among them, TWK10 has been chosen as the most favorable candidate for potential probiotic strain. TWK10 powder was prepared and provided by SYNBIO TECH INC. (Kaohsiung, Taiwan). Briefly, TWK10 was cultured in MRS broth at 37 °C for 16 h and harvested by centrifugation. The cell pellet was then freeze-dried and stored at −20 °C for further use. For the bacterial reverse mutation, chromosomal aberration, micronucleus, and 28-day subacute oral toxicity tests, TWK10 powder was prepared by mixing the freeze-dried bacterial cells with the appropriate amount of maltodextrin to a concentration of 1 × 10^10^ CFU/g and stored at −20 °C until use. 

### 2.2. Whole-Genome Sequencing and Taxonomic Identification

For 16S rRNA gene sequencing, the genomic DNA of TWK10 was extracted using the Genomic DNA Mini Kit (Geneaid Biotech, Taipei, Taiwan) according to the manufacturer’s instructions. PCR amplification involved an initial denaturation step at 94 °C for 5 min, followed by 30 cycles of 94 °C for 1 min, 50 °C for 1 min, and 72 °C for 1 min, with a final extension step at 72 °C for 8 min [24]. Next, 16S rRNA gene was amplified using universal primers (27F: 5’-AGAGTTTGATCCTGGCTCAG-3’ and 1492R: 5’-GGTTACCTTGTTACGACT-3’), and 16S rRNA gene sequence similarity was calculated using the BLAST program at NCBI (https://blast.ncbi.nlm.nih.gov/Blast.cgi: accessed on 3 December 2021). Phylogenetic analysis was conducted in MEGA X version 10.2.0 using the neighbor-joining method [25] with the Kimura two-parameter model [26,27]. The 16S rRNA gene sequences of reference strains (Figure S1) were downloaded from the NCBI database (https://www.ncbi.nlm.nih.gov/: accessed on 3 December 2021). For whole-genome sequencing, TWK10 genomic DNA was extracted using the Genomic-tip 20/G Kit from Qiagen (Hilden, Germany) and sequenced by Oxford Nanopore Technologies using the SQK-LSK109 Ligation Sequencing Kit on a MinION Flow Cell (R9.4.1) and Illumina MiSeq with paired-end (2 × 301 bp) mode. After decoding and refinement, the validated data were assembled using SPAdes software version 3.13.0 to obtain the genome sequence of TWK10. The genome sequences of reference bacterial strains were downloaded from the NCBI database. A phylogenomic tree based on the whole-genome sequences was reconstructed using the TYGS web server (https://tygs.dsmz.de/: accessed on 13 December 2021) [28]. The average nucleotide identity (ANI) values were estimated using orthologous average nucleotide identity (OrthoANI) [29] and digital DNA–DNA hybridization (dDDH) values were obtained using the Genome-to-Genome Distance Calculator (GGDC; http://ggdc.dsmz.de/ggdc.php#: accessed on 13 December 2021) with the recommended Formula 2 [30].

### 2.3. Annotation and Comparative Analysis of Whole-Genome Sequences 

Coding sequences (CDSs), transfer RNAs (tRNAs), ribosomal RNAs (rRNAs), and transfer-messenger RNAs (tmRNAs) were predicted and annotated using Prokka 1.14.6 software [31]. For functional prediction, the genome sequence of TWK10 was queried against locally installed eggNOG database v5 [32] using eggNOG-mapper tool v2.1.3 [33]. The circular map of the assembled genome of TWK10 was visualized using the web-based CGview program (http://cgview.ca/: accessed on 13 January 2022). The genome of TWK10 was uploaded to the Rapid Annotation using Subsystem Technology (RAST) Prokaryotic Genome Annotation Server (http://rast.nmpdr.org/: accessed on 13 January 2022) for annotation using the RASTtk pipeline. The sequence of the complete genome of TWK10, consisting of one chromosome and four plasmids, was deposited at NCBI (BioProject PRJNA800597 and Biosample SAMN25258448). For comparative genomic analysis, the genome sequences of two *L*. *plantarum* strains [ATCC 14917^T^ (RefSeq assembly accession number GCF_000143745.1) and WCFS1 (GCF_000203855.3)] were obtained from the NCBI database and used as references. The annotation files (GFF3) of these strains were generated using Prokka 1.14.6 software and were employed for pan-genome analysis using Roary software version 3.11.2 [34]. A Venn diagram of the unique/shared gene content was generated with a custom R script using the VennDiagram R package (https://cran.r-project.org/web/packages/VennDiagram/index.html: accessed on 13 January 2022). Multiple whole-genome alignments were performed using Mauve software (progressive MAUVE algorithm), which was designed for the identification and alignment of conserved genomic DNA in the presence of rearrangements [35].

### 2.4. Bioinformatic Analysis of Virulence Factors, Toxin-Related Genes, and Antimicrobial Resistance Genes

Virulence factor (VF)- and toxin-related genes were searched in the genome of TWK10 based on the virulence factor database (VFDB) [36], the pathogenicity island database (PAIDB) [37], and the VirulenceFinder and PathogenFinder datasets from the Center for Genomic Epidemiology (CGE) (https://cge.cbs.dtu.dk/services/VirulenceFinder/; https://cge.cbs.dtu.dk/services/PathogenFinder/: accessed on 17 January 2022). The BLASTn (ftp://ftp.ncbi.nlm.nih.gov/blast/: accessed on 17 January 2022) algorithm was used to predict putative VFs with >80% identity and >60% coverage. The sequences of proteins associated with microbial biogenic amine (BA) synthesis genes, including histidine decarboxylase (EC 4.1.1.22), tyrosine decarboxylase (EC 4.1.1.25), ornithine decarboxylase (EC 4.1.1.17), lysine decarboxylase (EC 4.1.1.18), and agmatine deiminase (EC 3.5.3.12), were obtained from UniProt (https://www.uniprot.org/: accessed on 17 January 2022) and constructed for the BLAST database. The BLASTp algorithm was used to predict the BA-producing ability of TWK10 employing >60% identity, >70% query coverage, and an e-value of <1.0e–20 as parameters. Antimicrobial resistance (AMR) genes in the genome were searched against the Comprehensive Antibiotic Resistance Database (CARD) (https://card.mcmaster.ca/: accessed on 17 January 2022) [38] using RGI software (DIAMOND homolog detection). The AMRFinder [39] and ResFinder [40] databases were used to detect AMR genes applying the default criteria. The Antibiotic Resistance Gene-Annotation (ARG-ANNOT) database [41] was used to search AMR genes using the BLASTn algorithm employing >80% identity and >60% coverage as parameters. The PHAge Search Tool (PHAST; http://phast.wishartlab.com/: accessed on 26 January 2022) [42] and PlasmidFinder 2.1 (https://cge.cbs.dtu.dk/services/PlasmidFinder/: accessed on 26 January 2022) [43] were used to search the phages and plasmids. MobileElementFinder database (v1.0.2) (https://cge.cbs.dtu.dk/services/MobileElementFinder/: accessed on 26 January 2022) [44] was also used to identify mobile genetic elements (MGEs) and their relation to antimicrobial resistance genes and virulence factors. 

### 2.5. Hemolytic Activity

The hemolytic activity of TWK10 was determined by streaking an overnight culture of TWK10 on blood agar supplemented with 5% (*w*/*v*) defibrinated sheep blood. The α-hemolytic *Streptococcus pneumoniae* ATCC 6305 [45] and the β-hemolytic *Staphylococcus aureus* ATCC 25923 [46] were used as positive control strains. The agar plates were incubated at 37 °C under aerobic conditions for 48 h following which the hemolytic activity of TWK10 was assessed based on the zone of hemolysis around the colonies [α-hemolysis—green-hued halo; β-hemolysis—clear halo; and γ-hemolysis—no change in color (no hemolytic activity)].

### 2.6. Antimicrobial Susceptibility Test

Minimal inhibitory concentrations (MICs) were determined according to the method described in ISO 10932:2010 in accordance with EFSA recommendations [47]. TWK10 was assessed for susceptibility to seven antibiotics (ampicillin, gentamicin, kanamycin, erythromycin, clindamycin, tetracycline, and chloramphenicol) using a 96-well plate gradient-dilution method. The plate was incubated anaerobically at 28 °C for 48 h and the optical density was observed at the wavelength of 625 nm using a Sunrise Basic ELISA plate reader (Tecan, Grödig, Austria). 

### 2.7. Bacterial Reverse Mutation Test (Ames Test)

Mutagenicity tests were conducted using five *Salmonella enterica* subsp. *enterica* serovar Typhimurium (*S.* Typhimurium) strains (TA98, TAl00, TA102, TA1535, and TA1537) obtained from MOLTOX Inc. (Boone, NC, USA). The plate incorporation test was performed both in the presence and absence of metabolic activation (S9 mix) according to procedures described in the safety assessment guideline of Health Food (TFDA, Taiwan: 1999; https://www.fda.gov.tw/ENG/index.aspx: accessed on 15 November 2020) and Guideline Test No. 471 of the Organization for Economic Co-operation and Development (OECD) [48] and each strain was tested in seven groups (a negative control group (sterile water), a positive control group, and five TWK10 treatment groups (5.000, 2.500, 1.250, 0.6250, and 0.3125 mg of TWK10/plate)). All groups were tested in triplicate. Briefly, 2 mL of soft agar (containing 200 μL of 0.5 mM histidine/biotin) was melted, to which 0.1 mL of an overnight bacterial suspension (1 × 10^9^ CFU/mL), 0.1 mL of a TWK10 or control solution, and 0.5 mL of S9 mix (if necessary) was added. After mixing, the sample was poured onto minimal glucose agar plates and incubated for 72 h at 35 °C. The number of spontaneous revertant colonies was then counted per plate and compared with that of the negative control. A sample was considered positive when (i) the number of revertant colonies was significantly higher (*p* < 0.05, one-way ANOVA) than that in the negative control group, (ii) the number of revertant colonies was at least two-fold higher than that in the negative control group, and (iii) a positive dose-response was recorded.

### 2.8. In Vitro Mammalian Cell Chromosomal Aberration Test

The Chinese hamster ovary (CHO)-K1 cell line was obtained from the Food Industry Research and Development Institute (FIRDI, Hsinchu, Taiwan). To assess TWK10 cytotoxicity, an in vitro chromosomal aberration assay was performed according to the procedures described in both the safety assessment guidelines for Health Food (TFDA, Taiwan: 1999) and Guideline Test No. 473 of the OECD [49]. Briefly, CHO-K1 cells were seeded in 6 cm dishes (1 × 10^6^/well) for 24 h. TWK10 powder was dissolved in the culture medium and used at concentrations of 5.00, 2.50, and 1.25 mg/mL. Cyclophosphamide monohydrate (CPP) (25 μg/mL) and mitomycin C (2.5 μg/mL) were used as the positive control reagents in the presence and absence of S9 mix, respectively. Ham’s F-12 medium with 10% fetal bovine serum was used as the negative control. In the absence of S9 mix, all the groups were treated for a short period (3 h) or continuously (20 h), while in the presence of S9 mix, all the groups were treated for a short period (3 h) only. After incubation, metaphase cells were collected and fixed on slides for Diff-Quik staining. For each treatment group, the incidence of chromosomal aberrations was scored for 100 metaphase cells. A response was considered positive if more than 3% of cells had chromosomal insults. Data are presented as the average number of chromosomal aberrations in 200 scored metaphase cells in duplicate measurements.

### 2.9. Rodent Peripheral Blood Micronucleus Test

This test was conducted according to procedures described in both the safety assessment guidelines for Health Food (TFDA, Taiwan: 1999) and Guideline Test No. 474 of the OECD [50]. All animal handling procedures were reviewed and approved by the Institutional Animal Care and Use Committee (IACUC) of Super Laboratory Co., Ltd. (New Taipei City, Taiwan) (approval no.108-11e). A total of 25 6-week-old male ICR mice (BioLASCO Co., Ltd., Taipei, Taiwan) were housed under controlled conditions with a 12:12 h light/dark cycle and randomly assigned to five groups, namely, a negative control group, a positive control group, and three TWK10 test groups (500, 1000, and 2000 mg/kg body weight). The test substance was administered once by oral gavage. CPP was given by intraperitoneal injection as the positive control. After 48 and 72 h of administration, 5-μL samples of peripheral blood were collected from the submandibular gland. Whole blood samples were dropped onto acridine orange-coated slides and incubated at room temperature for 3 h away from light. The percentage of reticulocytes (RETs) in every 1000 red blood cells (RBCs) (RETs/1000 RBCs) per animal and the incidence of micronuclei (Mn) in 1000 reticulocytes (Mn-RETs/1000 RETs) were determined under a fluorescence microscope.

### 2.10. Repeated Dose 28-Day Subacute Oral Toxicity Study in the Rat

This test was conducted according to procedures described in both the safety assessment guidelines for Health Food (TFDA, Taiwan: 1999) and Guideline Test No. 407 of the OECD [51]. Sprague–Dawley (SD) rats aged 6 weeks were obtained from BioLASCO Co., Ltd. Male and female rats were randomly assigned to four groups of 20 rats each, 10 rats per gender, and housed under controlled conditions (12:12 h light/dark cycle; temperature: 22 ± 3 °C; relative humidity: 55 ± 15%). All animal handling procedures were reviewed and approved by the IACUC of Super Laboratory Co., Ltd. (approval no. 108-9g). TWK10 was administered daily by oral gavage at the doses of 0, 500, 1000, and 2000 mg/kg body weight for 28 days. After overnight fasting, the rats were euthanized using CO_2_ inhalation. Blood samples were collected for the assessment of hematological and serum biochemical parameters. Hematological parameters, including white blood cell (WBC), RBC, platelet, neutrophil, lymphocyte, monocyte, eosinophil, and basophil counts, hemoglobin (Hb) levels, hematocrit (Hct), mean corpuscular volume (MCV), mean corpuscular hemoglobin (MCH), and mean corpuscular hemoglobin concentration (MCHC), were analyzed using an automated hematology analyzer (XT-1800i, Sysmex Corporation, Kobe, Japan). Prothrombin time (PT) was analyzed using a CA-1500 Blood Coagulation Analyzer (Sysmex). For serum biochemical analysis, blood glucose, blood urea nitrogen (BUN), creatinine, aspartate aminotransferase (AST), alanine aminotransferase (ALT), total protein, albumin, alkaline phosphatase (ALP), gamma-glutamyl transferase (γ-GT), cholesterol, triglyceride, calcium, phosphorus, sodium, potassium, chloride, globulin, and total bilirubin contents were analyzed using an automated chemistry analyzer (7070 Autoanalyzer, Hitachi, Tokyo, Japan).

### 2.11. Statistical Analysis

Data are expressed as means ± SD. Statistical analyses were performed using GraphPad Prism 7.04 (GraphPad Software, San Diego, CA, USA). For comparisons among multiple groups, data were analyzed by one-way ANOVA with Tukey’s or a Dunnett (for the Ames test) post-hoc test. The Kruskal–Wallis test with Dunn’s post-hoc test was used for multiple non-parametric comparisons. *p*-values < 0.05 were considered significant.

## 3. Results and Discussion

### 3.1. Taxonomic Identification of TWK10 

Accurate taxonomic characterization to the species level is the first step in the safety assessment of a probiotic strain. Therefore, the molecular-based identification was conducted to identify TWK10 at the species level. The phylogenetic analysis of 16S rRNA gene sequences showed that TWK10 belonged to the genus *Lactiplantibacillus* (Figure S1). 16S rRNA gene sequence similarities between TWK10 and each of its closest neighbors (*L. plantarum* subsp. *plantarum* ATCC 14917^T^*, L. argentoratensis* DSM 16365^T^, *L. paraplantarum* DSM 10667^T^, and *L. pentosus* DSM 20314^T^) were high, with values of over 99.7%. The values for ANI between TWK10 and each of its closest neighbors were 99.12%, 95.55%, 86.10%, and 79.89%, respectively, while the dDDH values were 92.7%, 62.9%, 31.6%, and 24.4%, respectively (Table S1). The phylogenomic tree based on whole-genome sequences showed that TWK10 and *L. plantarum* subsp. *plantarum* ATCC 14917^T^ formed an independent cluster (Figure S2). ANI and dDDH values of 95–96% and 70%, respectively, correspond to a DDH value of 70% and are the most widely used as boundaries in species delineation. Thus, based on these genotypic results, TWK10 was identified as *Lactiplantibacillus plantarum*.

### 3.2. Genome Structure and General Features of TWK10 

The assembled genome of TWK10 (3,273,862 bp) comprised a circular chromosome of 3,205,949 bp and four circular plasmids ranging in size from 7034 to 32,685 bp. The overall G + C content of the chromosome was 44.4%, whereas that of the plasmids was slightly lower, ranging from 35.3% to 39.4%. A total of 3173 genes were predicted by Prokka, including 3088 protein-coding genes (CDSs), 16 rRNA-coding genes, 68 tRNA-coding genes, and 1 tmRNA-coding gene (Figure S3 and Table 1). For functional classification, the TWK10 genome was analyzed using the clusters of orthologous genes (COG) database (http://www.ncbi.nlm.nih.gov/COG/: accessed on 10 January 2022), resulting in the annotation of 2582 genes. Of the 3266 predicted COGs in TWK10, 684 (20.9%) coded for hypothetical proteins with no functional assignment.

Of the remaining 2582 predicted COGs in TWK10, 621 (24.1%) were classified into category S (Function unknown). Meanwhile, the major COG categories were “Transcription” (category-K, 276 genes), “Replication, recombination and repair” (category-L, 240 genes), “Carbohydrate transport and metabolism” (category-G, 175 genes), “Translation, ribosomal structure and biogenesis” (category-J, 171 genes), “Amino acid transport and metabolism” (category-E, 163 genes), and “Cell wall/membrane/envelope biogenesis” (category-M, 157 genes). The number of genes categorized into “Replication, recombination and repair” (category-L)” in TWK10 was greater than that in ATCC 14917^T^ and WCFS1 (Table S2). Additionally, TWK10 genes were categorized into 27 feature types and 231 subsystems according to SEED subsystem categorization. Among these subsystems, “Carbohydrates” was the most represented subsystem (230 genes), followed by “Amino Acids, Lipids, and Isoprenoids” (173 genes), “Protein Metabolism” (136 genes), and “Cofactors, Vitamins, Prosthetic groups, Pigments” (107 genes). The category of “Virulence, Disease, and Defense” included genes involved in “Invasion and intracellular resistance” (9 genes) and “Resistance to antibiotics and toxic compounds” (29 genes). The numbers of genes categorized into “Carbohydrates”, “Cell Wall and Capsule”, and “Respiration” in WFCS1 were 248, 77, and 16, respectively, values that were greater than those in TWK10. These annotation results demonstrated that the distribution of COG functional categories and SEED subsystem functions in the TWK10 genome was similar to that in the two reference genomes (Table S3). However, a total of 11 and 31 strain-specific genes were found within TWK10 and WCFS1, respectively. In particular, six genes classified as SEED subsystem functions, “rhamnose containing glycans” and “sialic acid metabolism”, which are thought to be involved in cell adhesion, and nine genes involved in the metabolism of carbohydrates were characteristic in the WCFS1 genome, whereas three genes categorized in SEED subcategory “Phages, Prophages” were found in the TWK10 genome (Table S4).

### 3.3. Comparative Genomic Analysis

Next, the genome of TWK10 was compared with the two reference genomes [WCFS1, a model probiotic strain [52,53] and ATCC 14917^T^, a type strain of *L. plantarum*] using Roary and Mauve software. A total of 2531 genes were found to be conserved among the three genomes. In total, 88.5% and 83.7% of the genes in the ATCC 14917^T^ and WCSF1 strains, respectively, were orthologous to TWK10 genes based on results from the Roary workflow. Moreover, 351 (12.8%), 224 (7.5%), and 387 (12.4%) of genes were found to be specific to TWK10, ATCC 14917^T^, and WCFS1, respectively. TWK10 shared 123 and 84 genes with ATCC 14917^T^ and WCFS1, respectively (Figure S4A). Whole-genome alignment of the three strains using a progressive MAUVE algorithm revealed that the genomes of TWK10 and WCFS1 had strain-specific regions, and most of the local collinear blocks (LCBs) were shared among the three strains (Figure S4B). In summary, the genome of TWK10 exhibited high similarity to the genomes of ATCC 14917^T^ and WCFS1. Prophages were widely distributed among probiotic strains commonly used in dairy fermentation [54]. The prophages may facilitate horizontal gene transfer to disseminate the antimicrobial resistance genes via phage-mediated transduction to other bacteria. Therefore, the presence of the prophages on the TWK10 genome was analyzed using PHAST database, and five prophage regions were identified as three intact phages and two incomplete phages, respectively (Table S5). Further detailed comparative genomic analyses along with WCFS1, a model of probiotic strain, are still needed not only to elucidate the safety and the characteristic probiotic effects of TWK10, but also to clarify the difference between TWK10 and WCFS1.

### 3.4. Determination of Virulence Factors and Toxin-Related Genes

VFs refer to elements (i.e., gene products) that allow a microorganism to colonize a host niche, proliferate, and cause tissue damage or systemic inflammation. VFs include secreted proteins such as protein toxins and enzymes as well as cell-surface structures such as capsular polysaccharides, lipopolysaccharides, and outer-membrane proteins that directly contribute to disease processes [36]. To identify known VFs and toxin-related genes, and hence pathogenicity, all the annotated genes of TWK10 were analyzed using BLASTn and the VFDB, PAIDB, and CGE databases. No putative virulence genes were identified under the stringent criteria of >80% sequence identity and >60% query coverage. 

### 3.5. Hemolytic Activity

Blood agar containing general nutrients and 5% sheep blood is a useful medium for determining the hemolytic capability of microorganisms. In this study, TWK10 and two reference strains (*S. pneumoniae* ATCC 6305 and *S*. *aureus* ATCC 25923) were streaked on sheep blood agar plates and incubated at 37°C for 48 h for the determination of hemolytic activity. As shown in Figure S5, TWK10 exhibited no hemolytic activity, whereas *S. pneumoniae* ATCC 6305 and *S. aureus* ATCC 25923 displayed α-hemolytic and β-hemolytic activity, respectively.

### 3.6. Antimicrobial Resistance and Associated Genes

The antimicrobial susceptibility of TWK10 was determined by measuring the MICs for seven antibiotics (ampicillin, gentamicin, kanamycin, erythromycin, clindamycin, tetracycline, and chloramphenicol). The results were compared to the cut-off values for *L. plantarum*/*pentosus* as defined by the EFSA. The obtained MIC values indicated that TWK10 was susceptible to all the antibiotics, except kanamycin. The MIC for kanamycin (128 mg/L) was 2-fold higher than the cut-off value specified by the EFSA (Table 2). This result was consistent with those of previous studies [55,56,57]. Many *Lactobacillus* species are resistant to aminoglycoside antibiotics, such as streptomycin, kanamycin, and gentamicin [58]. In addition, the enzyme aminoglycoside phosphotransferase type III, encoded by the *aph*(*3”*)*-III* gene, which is associated with resistance to kanamycin, has previously been detected in kanamycin-resistant *L. plantarum* strains [55,57]. Moreover, Feng et al. [59] demonstrated that non-synonymous mutations in genes regulating major facilitator family protein, ABC transporter substrate-binding protein, and histidine kinase play an important role in increasing kanamycin resistance in *L. plantarum*. When a bacterial strain demonstrates resistance to a specific antibiotic, the antimicrobial resistance determinants must be identified [47]. Accordingly, we undertook a genome-wide search for AMR genes in the TWK10 genome using the CARD, AMRFinder, ResFinder, and ARG-ANNOT databases. No genes associated with resistance to ampicillin, gentamicin, kanamycin, erythromycin, clindamycin, tetracycline, or chloramphenicol were found in the genome of TWK10 (Table 2). This result indicates that kanamycin resistance in TWK10 is unlikely to be transferred to other bacteria via horizontal gene transfer (HGT) and can be considered safe regarding antimicrobial resistance. Based on the MobileElementFinder database searching, a total of 11 insertion sequences (ISs) on the chromosome of TWK10 were identified as *ISP2* (a member of IS1182 family), and *ISLsa1* and *ISLpl1* (members of the IS30 family), respectively (Table S6). We have confirmed that no antimicrobial resistance genes on the prophage regions on the genome of TWK10. This implies there is no risk of HGT of antimicrobial resistance genes between TWK10 and other bacteria.

### 3.7. BA Production

Biogenic amines (BAs) in foods are of great concern in terms of both food spoilage and food safety. BAs are mainly produced by microbial-mediated decarboxylation of amino acids, particularly tyrosine, histidine, and tryptophan to tyramine, histamine, and tryptamine, respectively, and some *Lactobacillus* strains can produce biogenic amines via amino acid decarboxylase activity [60,61,62,63]. A search of the TWK10 genome for genes related to adverse metabolite production did not identify any genes associated with the production of BAs, including genes coding for the enzymes histidine decarboxylase (EC 4.1.1.22), tyrosine decarboxylase (EC 4.1.1.25), ornithine decarboxylase (EC 4.1.1.17), lysine decarboxylase (EC 4.1.1.18), and agmatine deiminase (EC 3.5.3.12), which are responsible for the synthesis of BAs in lactic acid bacteria [64].

### 3.8. Analysis of Genotoxicity

The Ames test, chromosomal aberration test, and peripheral blood micronucleus test were performed to assess the genotoxicity of TWK10. The mutagenicity of TWK10 was evaluated against five *S.* Typhimurium strains (TA98, TA100, TA102, TA1535, and TA1537) using a bacterial reverse mutation assay with different doses of TWK10. Compared with the negative control, no significant increase in the number of revertant colonies was observed for the different *S.* Typhimurium strains following treatment with different concentrations of TWK10, both in the presence and absence of S9 mix; in contrast, a significant (*p* < 0.05) increase in the number of revertant colonies was observed in the positive controls (Table 3). These results indicated that TWK10 is not mutagenic toward histidine-auxotrophic *S.* Typhimurium strains.

In the chromosomal aberration test, CHO-Kl cells were co-cultured with TWK10 at the doses of 1.25, 2.50, and 5.00 mg/mL, after which the number of cells with abnormal chromosomes was calculated. For the positive control groups treated with CPP in the presence of S9 mix or mitomycin C in the absence of S9 mix, the total number of cells with chromosomal aberrations (CAs) ranged from 12.5% to 13.5%. In the absence of S9 activation, the frequency of CAs in CHO-K1 cells treated with 1.25–5.00 mg/mL TWK10 after 3 and 20 h of incubation ranged from 1% to 2%, while those of the positive controls increased in 12.5%. In the presence of S9 activation after 3 h of incubation, the number of cells with an abnormal chromosome ranged from 1.5% to 2%, while for the positive control this number increased to 13.5%. For the negative control groups, the number of cells with chromosomal aberrations ranged from 1% to 2% (Table 4). These results indicated that the frequency of chromosomal aberrations in all experimental conditions was below 2%, suggesting that TWK10 is safe in terms of genotoxicity at doses of up to 5 mg/mL.

The percentage of reticulocytes in the total number of red blood cells and the incidence of micronuclei in ICR mice administered TWK10 at the doses of 500, 1000, and 2000 mg/kg body weight were also determined. After 48 and 72 h of observation, the reticulocyte ratios (RETs/1000 RBCs) in the CPP-treated positive controls were 15% and 17%, respectively, and were significantly lower (*p* = 0.008) than those of the negative controls. The incidence of micronucleated reticulocytes (Mn-RETs/1000 RETs) in the positive control groups after 48 and 72 h of observation was significantly higher when compared with that for the negative control and TWK10 treatment groups. In contrast, TWK10 treatment did not affect either the reticulocyte ratio or the micronucleus incidence in mice at any of the doses tested, and in the negative control groups (Table 5). These results indicated that TWK10 is non-mutagenic. 

### 3.9. Repeated Dose 28-Day Subacute Oral Toxicity Test

TWK10 was administered orally to SD rats daily at the doses of 500, 1000, and 2000 mg/kg of body weight for 28 days. No abnormal symptoms were observed in general behavior and physical activity either in the control or TWK10 treatment groups. During the 28-day trial, the body weight of rats in the treatment groups was recorded every week. Although the body weight of each group increased, no significant difference was observed between the genders in the TWK10 treatment groups and the control group. After 28 days of repeated oral administration of TWK10, the rats were euthanized and the organs, including the testes/ovaries, adrenal glands, spleen, kidneys, heart, brain, and liver, were carefully collected for weight measurement and histological assessment. Male rats in the low-dose TWK10 group showed significantly lower (*p* < 0.05) brain weight relative to the control group. However, no dose-dependent effect on brain weight was recorded, and no correlative histopathological lesions were observed. Accordingly, this alteration was not considered adverse. In female rats, no significant difference in organ weight was observed between the control and TWK10 treatment groups (Table 6). Furthermore, there was no significant change in appearance, behavior, or survival rate during the 28-day trial.

After 28 consecutive days of TWK10 administration, blood samples were collected from rats for hematological and biochemical analysis. TWK10 administration did not cause significant alterations in most of the hematological parameters assessed. The eosinophil count in male rats was significantly lower (*p* < 0.05) in the low-dose TWK10 group than in the control group, while the monocyte count in female rats administered medium and high doses of TWK10 was significantly lower (*p* < 0.05) than that in female rats of the control group (Table S7). In male rats, the levels of creatinine in the medium- and high-dose TWK10 groups and the albumin content in the medium-dose TWK10 group were significantly lower (*p* < 0.05) than those of the control group. In female rats, the levels of creatinine in the medium- and high-dose TWK10 groups and those of ALT in the high-dose TWK10 group were significantly lower (*p* < 0.05) than those in the control group (Table S8). However, the observed differences in hematological and biochemical parameters were not dose-dependent, and all the values were within the normal range. Consequently, these findings were not considered to be adverse or treatment-related.

## 4. Conclusions

In this study, the safety of TWK10 was comprehensively assessed using both in vitro and in vivo approaches. These included whole-genome sequence analysis, assessment of hemolytic activity, an antimicrobial susceptibility test, the Ames bacterial reverse mutation assay, the chromosomal aberration test in CHO-K1 cells, and a peripheral blood micronucleus test in ICR mice. The results demonstrated that TWK10 is both non-hemolytic and, except for kanamycin, susceptible to all the antibiotics that are prescribed for *L. plantarum* by the EFSA. Additionally, TWK10 has no mutagenic or genotoxic potential. The 28-day repeated-dose subacute oral toxicity test in SD rats confirmed that TWK10 has no subacute toxicity at the dosage of 2000 mg/kg body weight/day. Furthermore, based on a comparative genomic analysis, we found that the genome of TWK10 is generally consistent with that of *L. plantarum* WCFS1 which has been confirmed to be safe as a probiotic strain by genome analysis, and contains no antimicrobial resistance genes (including for kanamycin), VFs, toxin-related genes, genes encoding BAs, or transferable antibiotic resistance genes. Based on the lack of treatment-related adverse effects at the highest orally administered dose of TWK10, our findings clearly demonstrate that TWK10 has high potential of being safe for human consumption as a probiotic. Further studies in clinical trials and long-term administration in humans are needed not only to validate the safety of TWK10 obtained in this study, but also to verify the health-promoting benefits confirmed in our previous studies, such as enhancing exercise performance, promoting increases in muscle mass and strength, and attenuating age-associate cognitive decline.

## Figures and Tables

**Table 1:** Comparison of the genomic features of *Lactiplantibacillus plantarum* strains TWK10, ATCC 14917^T^, and WCFS1.

	Strain		
	TWK10	ATCC 14917^T^	WCFS1
Accession No.		GCA_000143745.1	GCA_000203855.3
Genome size (bp)	3,273,862	3,212,261	3,348,624
G + C content (%)	44.4	44.5	44.4
No. of contigs	5	9	4
N50 length (bp)	3,205,949	152,365	3,308,273
L50	1	6	1
Genes	3173	3061	3211
CDS	3088	2999	3123
tRNA	68	59	71
rRNA	16	2	16
tmRNA	1	1	1

Bp, base pairs; CDS, coding sequence; tRNA, transfer RNA; rRNA, ribosomal RNA; tmRNA, transfer-messenger RNA.

**Table 2:** The antimicrobial sensitivity of TWK10.

Antibiotics	MIC ^a^	Cut-Off Value ^b^	Sensitivity ^c^	Antimicrobial Resistance Gene
Ampicillin	<0.5	2	S	ND ^d^
Gentamicin	16	16	S	ND
Kanamycin	128	64	R	ND
Erythromycin	<0.5	1	S	ND
Clindamycin	2	4	S	ND
Tetracycline	16	32	S	ND
Chloramphenicol	4	8	S	ND

^a^ MIC, minimal inhibitory concentration (mg/L) toward TWK10. ^b^ Microbiological cut-off values (mg/L) for antibiotics recommended by the European Food Safety Authority. ^c^ S, Susceptible; R, Resistant. ^d^ ND, not detected.

**Table 3:** Ames test results for TWK10 using *S.* Typhimurium strains.

	Number of Revertant Colonies/Plate				
	*S.* Typhimurium Strain				
	TA98	TA100	TA102	TA1535	TA1537
**With S9** **mix**					
Negative control ^a^	25 ± 5	211 ± 8	428 ± 16	16 ± 4	9 ± 3
Positive control ^b^	388 ± 41 *	650 ± 11 *	859 ± 40 *	284 ± 22 *	303 ± 14 *
TWK10 (5.0000 mg)	23 ± 1	202 ± 9	425 ± 27	14 ± 1	7 ± 1
TWK10 (2.5000 mg)	27 ± 2	210 ± 5	423 ± 9	17 ± 4	9 ± 2
TWK10 (1.2500 mg)	31 ± 2	212 ± 7	421 ± 25	17 ± 5	10 ± 2
TWK10 (0.6250 mg)	28 ± 4	212 ± 7	447 ± 32	18 ± 1	10 ± 3
TWK10 (0.3125 mg)	28 ± 2	217 ± 1	430 ± 19	19 ± 7	14 ± 3
**Without S9** **mix**					
Negative control ^a^	21 ± 3	171 ± 7	371 ± 26	17 ± 3	9 ± 2
Positive control ^c^	265 ± 20 *	616 ± 52 *	770 ± 24 *	221 ± 12 *	245 ± 18 *
TWK10 (5.0000 mg)	22 ± 1	175 ± 6	368 ± 31	20 ± 1	8 ± 3
TWK10 (2.5000 mg)	25 ± 1	178 ± 16	373 ± 5	17 ± 1	8 ± 3
TWK10 (1.2500 mg)	29 ± 3	179 ± 11	358 ± 24	18 ± 2	7 ± 2
TWK10 (0.6250 mg)	28 ± 4	185 ± 15	359 ± 29	15 ± 1	8 ± 2
TWK10 (0.3125 mg)	29 ± 2	169 ± 18	375 ± 16	21 ± 2	8 ± 3

Data are presented as means ± SD (*n* = 3). Statistically significant differences between the number of revertant colonies of each column and that of the negative control were calculated using one-way ANOVA with Dunnett’s post hoc test. The asterisk (*) indicates a significant difference relative to the negative control (*p* < 0.05). ^a^ Sterile water was used as a negative control. ^b^ Positive controls with S9 for TA98: benzo[α]pyrene, 4 μg/plate; for TA100: 2-aminoanthracene, 4 μg/plate; for TA102: 2-aminoanthracene, 10 μg/plate; for TA1535: 2-aminoanthracene, 4 μg/plate; for TA1537: 2-aminoanthracene, 4 μg/plate. ^c^ Positive controls without S9 for TA98: 4-nitroquinoline 1-oxide, 0.5 μg/plate; for TA100: sodium azide, 5.0 μg/plate; for TA102: mitomycin C, 0.5 μg/plate; for TA1535: sodium azide, 0.4 μg/plate; for TA1537: 9-aminoanthracene, 50.0 μg/plate.

**Table 4:** Chromosomal aberration test for TWK10 in CHO-K1 cells.

	Number of Chromosome Aberrations									Total Number of Chromosomal Aberrations (%)	Number of Cells with Chromosomal Aberrations (%)
	(Per 100 Cells)										
	G	B	D	R	g	b	Int	Itr	Other		
**3 h with S9 mix**											
Negative control ^a^	0	0	0	0	1	0	0	0	0	1.0	1.0
Positive control ^b^	2.5	1.5	0	0.5	4	2	0.5	0	2.5	13.5	13.5
TWK10 (5.00 mg/mL)	0	0	0	0.5	1	0	0	0	0	1.5	1.5
TWK10 (2.50 mg/mL)	0	0	0	0	1.5	0.5	0	0	0	2.0	1.5
TWK10 (1.25 mg/mL)	0	0	0	0	0.5	1	0	0	0	1.5	1.5
**3 h without S9 mix**											
Negative control ^a^	0	0	0	0	1.5	0.5	0	0	0	2.0	1.0
Positive control ^c^	2	0.5	0	0.5	4.5	2.5	0.5	0	2	12.5	12.5
TWK10 (5.00 mg/mL)	0	0.5	0	0	0.5	0	0	0	0.5	1.5	1.5
TWK10 (2.50 mg/mL)	0	0	0	0	1.5	0.5	0	0	0	2.0	2.0
TWK10 (1.25 mg/mL)	0	0	0	0	0.5	0.5	0	0	0	1.0	1.0
**20 h without S9 mix**											
Negative control ^a^	0	0	0	0.5	0.5	0	0	0	0	1.0	1.0
Positive control ^c^	2	0.5	0.5	0.5	4.5	2.5	0	0	2	12.5	12.5
TWK10 (5.00 mg/mL)	0	0	0	0	1.5	0.5	0	0	0	2.0	1.5
TWK10 (2.50 mg/mL)	0	0	0	0	0.5	0.5	0	0	1	2.0	1.5
TWK10 (1.25 mg/mL)	0.5	0.5	0	0	1	0	0	0	0	2.0	2.0

Data are the average of duplicate measurements. ^a^ Ham’s F-12 medium with 10% fetal bovine serum. ^b^ 25 μg/mL cyclophosphamide monohydrate was used with S9. ^c^ 2.5 μg/mL mitomycin C was used without S9. G, chromosome gap; B, chromosome break; D, dicentric; R, ring; g, chromatid gap; b, chromatid break; Int, interchange; Itr, intrachange; other, polyploidy cell and cell with endoreduplication or centromeric disruption.

**Table 5:** Reticulocyte ratio and micronucleus incidence in peripheral blood of ICR mice administered TWK10.

		Reticulocyte Ratio ^a^		Micronucleus Incidence ^b^	
		Median	*p*-Value	Median	*p*-Value
		(Q1–Q3) ^c^		(Q1–Q3) ^c^	
**48 h**					
Negative control ^d^		46 (42.5–46.5)	–	0 (0.0–1.0)	–
Positive control ^e^		15 (13.5–18.0)	0.008 *	17 (15.5–18.5)	0.008 *
TWK10 (500 mg/kg BW)		46 (41.0–49.0)	0.889	1 (0.0–1.5)	0.683
TWK10 (1000 mg/kg BW)		43 (41.0–48.5)	0.984	1 (0.0–1.5)	0.683
TWK10 (2000 mg/kg BW)		45 (41.5–45.5)	0.437	1 (0.5–1.5)	0.365
**72 h**					
Negative control ^d^		47 (46.5–48.5)	–	1 (0.0–1.5)	–
Positive control ^e^		17 (16.5–18.5)	0.008 *	15 (14.5–17.0)	0.008 *
TWK10 (500 mg/kg BW)		47 (45.5–48.0)	0.619	1 (0.0–1.5)	1.000
TWK10 (1000 mg/kg BW)		47 (46.0–48.5)	0.881	1 (0.5–1.5)	0.881
TWK10 (2000 mg/kg BW)		47 (45.5–47.5)	0.444	1 (0.5–1.5)	0.881

^a^ Reticulocyte ratio, reticulocytes (RETs)/1000 red blood cells (RBCs); *n* = 5. ^b^ Micronucleus incidence, reticulocytes with micronucleus (Mn-RET)/1000 reticulocytes (RETs); *n* = 5. ^c^ Q1, 25% quartile; Q3, 75% quartile. ^d^ Sterile water was used as the negative control. ^e^ Cyclophosphamide monohydrate at 50 mg/kg body weight served as the positive control. Statistically significant differences between the median values of each column and the negative control were calculated using the Kruskal–Wallis test with Dunn’s post hoc test. The asterisk (*) indicates a significant difference compared with the negative control (*p* < 0.05).

**Table 6:** Body weight and organ weight of Sprague–Dawley rats administered TWK10 for 28 days.

Male				
	Control	Low Dose (500 mg TWK10/kg BW)	Medium Dose (1000 mg TWK10/kg BW)	High Dose (2000 mg TWK10/kg BW)
Body weight (before)	207.6 ± 9.8	207.6 ± 8.9	207.6 ± 8.4	207.7 ± 7.7
Body weight (after)	377.8 ± 29.3	362.6 ± 29.7	370.9 ± 18.5	351.3 ± 25.6
Testis	3.236 ± 0.281	3.284 ± 0.251	3.325 ± 0.207	3.330 ± 0.184
Adrenal gland	0.075 ± 0.016	0.070 ± 0.014	0.073 ± 0.014	0.073 ± 0.014
Spleen	0.731 ± 0.086	0.677 ± 0.154	0.720 ± 0.118	0.661 ± 0.128
Kidney	3.438 ± 0.360	3.198 ± 0.280	3.396 ± 0.260	3.198 ± 0.327
Heart	1.528 ± 0.183	1.486 ±0.114	1.510 ± 0.130	1.435 ± 0.173
Brain	2.075 ± 0.062	1.971 ± 0.120 *	2.030 ± 0.063	2.024 ± 0.078
Liver	15.027 ± 1.653	13.803 ± 1.226	14.672 ± 1.132	14.194 ± 1.752
Female				
	Control	Low dose (500 mg TWK10/kg BW)	Medium dose (1000 mg TWK10/kg BW)	High dose (2000 mg TWK10/kg BW)
Body weight (before)	179.4 ± 8.0	178.0 ± 8.9	180.0 ± 8.6	181.0 ± 8.6
Body weight (after)	221.8 ± 16.5	232.1 ± 11.5	227.5 ± 20.8	232.9 ± 15.5
Ovary	0.117 ± 0.021	0.121 ± 0.034	0.121 ± 0.038	0.111 ± 0.024
Adrenal gland	0.073 ± 0.010	0.082 ± 0.010	0.074 ± 0.011	0.080 ± 0.010
Spleen	0.426 ± 0.088	0.514 ± 0.080	0.499 ± 0.074	0.509 ± 0.074
Kidney	1.952 ± 0.131	2.026 ± 0.127	2.083 ± 0.272	2.046 ± 0.171
Heart	0.980 ± 0.080	1.009 ± 0.081	0.985 ± 0.131	0.969 ± 0.093
Brain	1.921 ± 0.066	1.895 ± 0.073	1.927 ± 0.079	1.881 ± 0.083
Liver	8.512 ± 0.952	8.902 ± 0.943	8.718 ± 1.424	8.601 ± 0.848

Data are presented as means ± SD, *n* = 10. Statistically significant differences between the body weight and absolute organ weight of each group and those of the control group were calculated using one-way ANOVA with Tukey’s post-hoc test. The asterisk (*) indicates a significant difference relative to the control group (*p* < 0.05). BW, body weight.

## Data Availability

The datasets used and/or analyzed in the current study are available from the corresponding author on reasonable request.

## Supplementary Materials

The following supporting information can be downloaded at: https://www.mdpi.com/article/10.3390/microorganisms10040784/s1, Figure S1: Phylogenetic tree based on 16S rRNA gene sequences showing the relationship of strain TWK10 with type strains of closely related species in the genus *Lactiplantibacillus*; Figure S2: The phylogenomic tree based on whole genome sequences of TWK10 and its closely related the type strains in the genus *Lactiplantibacillus*; Figure S3: Genomic features of *Lactiplantibacillus plantarum* TWK10; Figure S4: Genomic comparison of the TWK10 with *L. plantarum* strains, ATCC 14917^T^ and WCFS1; Figure S5: Hemolytic activity of TWK10; Table S1: Average nucleotide identity (ANI) values and digital DNA–DNA hybridization (dDDH) prediction values between strain TWK10 and genetically closely related species in the genus *Lactiplantibacillus*; Table S2: Genes associated with general COG functional categories in TWK10 genome; Table S3: Comparison of SEED subsystem features of TWK10 and *L. plantarum* reference strains, ATCC14917^T^ and WCFS1; Table S4: Strain-specific SEED subsystem functions that differ between TWK10 and WCFS1; Table S5: Bioinformatic analysis of phage sequences in the genome of TWK10; Table S6: List of insertion sequences identified in the genome of TWK10; Table S7: Hematological parameters of male and female SD rats after oral administration of TWK10 for 28 days; Table S8: Serum biochemical parameters of male and female SD rats after oral administration of TWK10 for 28 days.
