# Supplementary material for: Safety Assessment of Lactiplantibacillus plantarum TWK10 Based on Whole-Genome Sequencing, Phenotypic, and Oral Toxicity Analysis
Source: Microorganisms. 2022 Apr 7;10(4):784. doi: 10.3390/microorganisms10040784 (PMC9031848; doi:10.3390/microorganisms10040784)
Supplement: Supplementary file 1 [file microorganisms-10-00784-s001.zip › microorganisms-1654501-supplementary.pdf]

microorganisms

MDPI

Supplementary materials

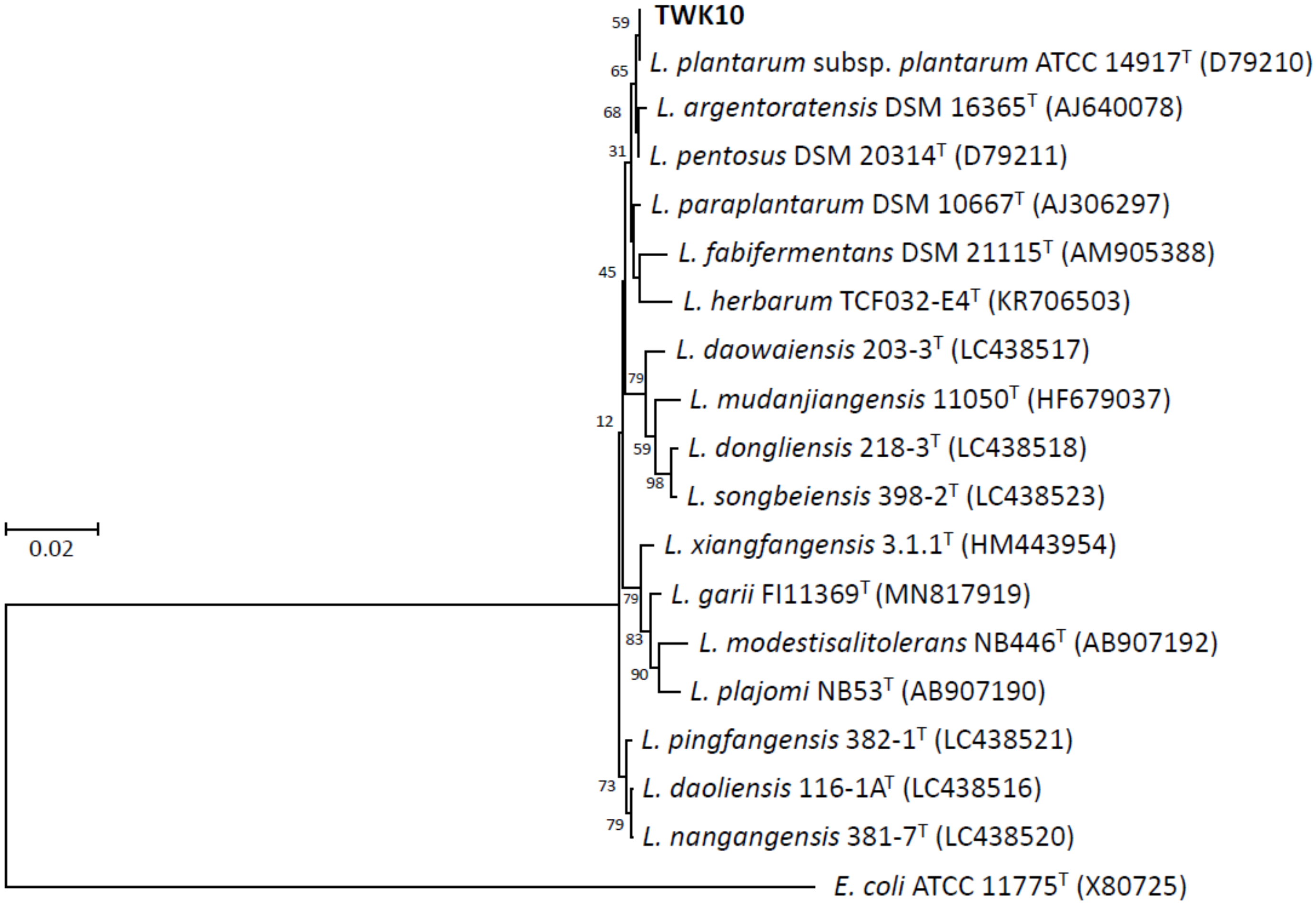

Figure S1. Phylogenetic tree based on 16S rRNA gene sequences showing the relationship of strain TWK10 with type strains of closely related species in the genus *Lactiplantibacillus*. The tree was reconstructed by the neighbor-joining method with Kimura's two-parameter model, and *Escherichia coli* ATCC 11775^T was used as an outgroup. Bootstrap values with 1000 replications are given at nodes. Bar, 2% sequence divergence.

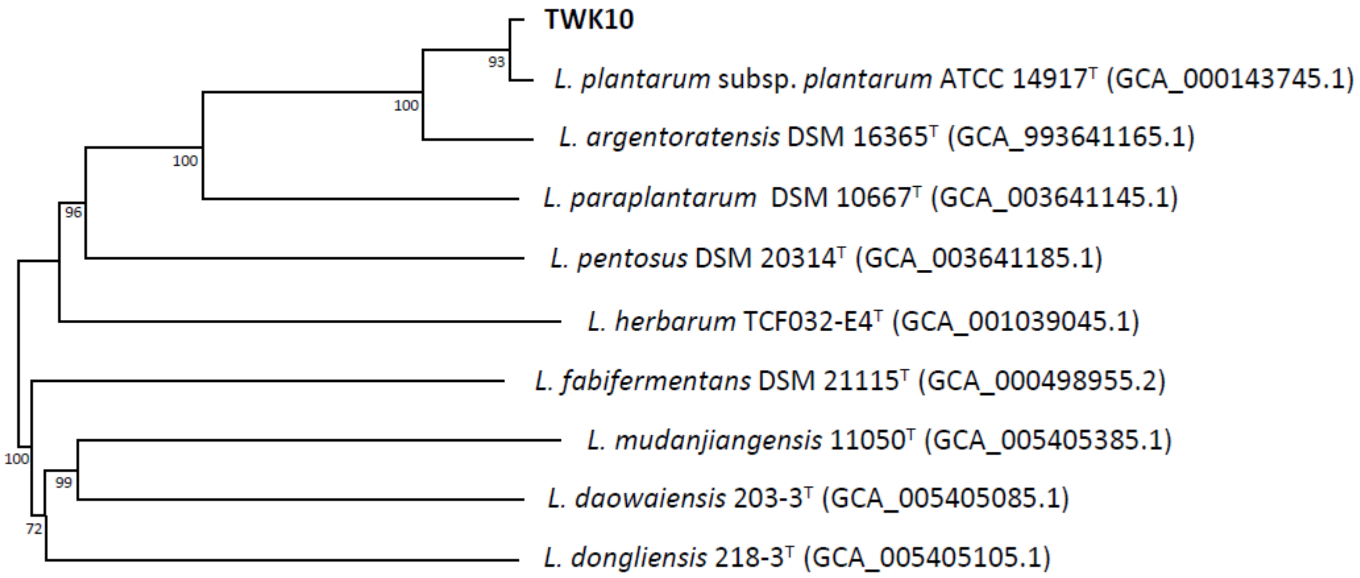

Figure S2. The phylogenomic tree based on whole genome sequences of TWK10 and its closely related the type strains in the genus *Lactiplantibacillus*. Tree inferred with FastME 2.1.6.1 from GBDP distances calculated from genome sequences. The branch lengths are scaled in terms of GBDP distance formula d_5. The numbers above branches are GBDP pseudo-bootstrap support values > 60 % from 100 replications, with an average branch support of 94.3 %.

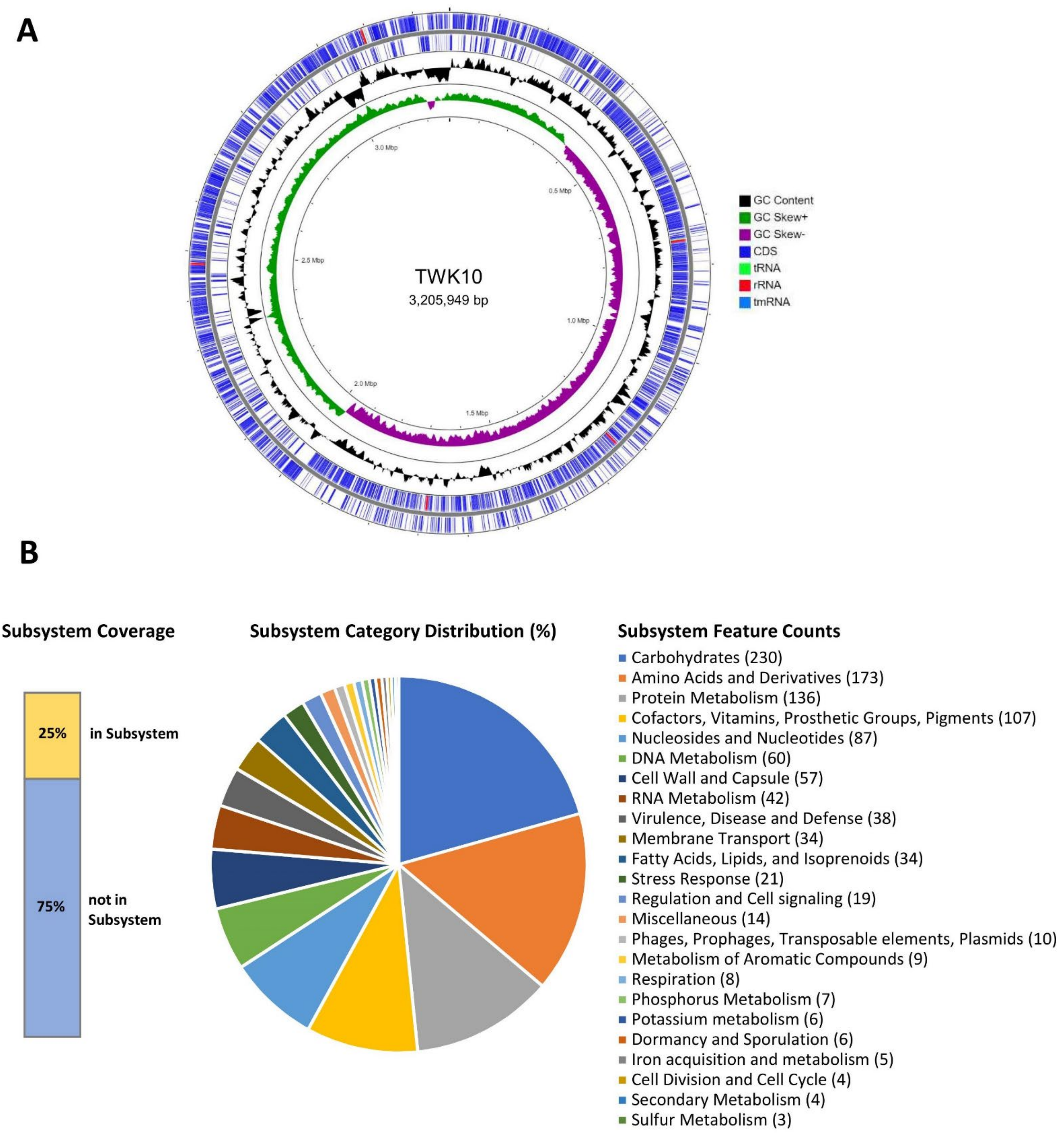

Figure S3. Genomic features of *Lactiplantibacillus plantarum* TWK10. (**A**) The outer two circles show the CDS, tRNA, rRNA, and tmRNA on the forward and reverse strands. The third circle shows the GC content (black). The fourth circle represents the GC skew (C – G)/(C + G) curve (positive GC skew, green; negative GC skew, violet). (**B**) The genome of *L. plantarum* TWK10 annotated using the Rapid Annotation System Technology (RAST) server. The orange-colored part (25%) in the bar chart corresponds to the percentage of proteins included. The pie chart demonstrates the percentage distribution of SEED subsystem features. Numerals in parentheses are the counts of genes in each subsystem feature. CDS: coding sequence; tRNA: transfer RNA; rRNA: ribosomal RNA; tmRNA: transfer-messenger RNA.

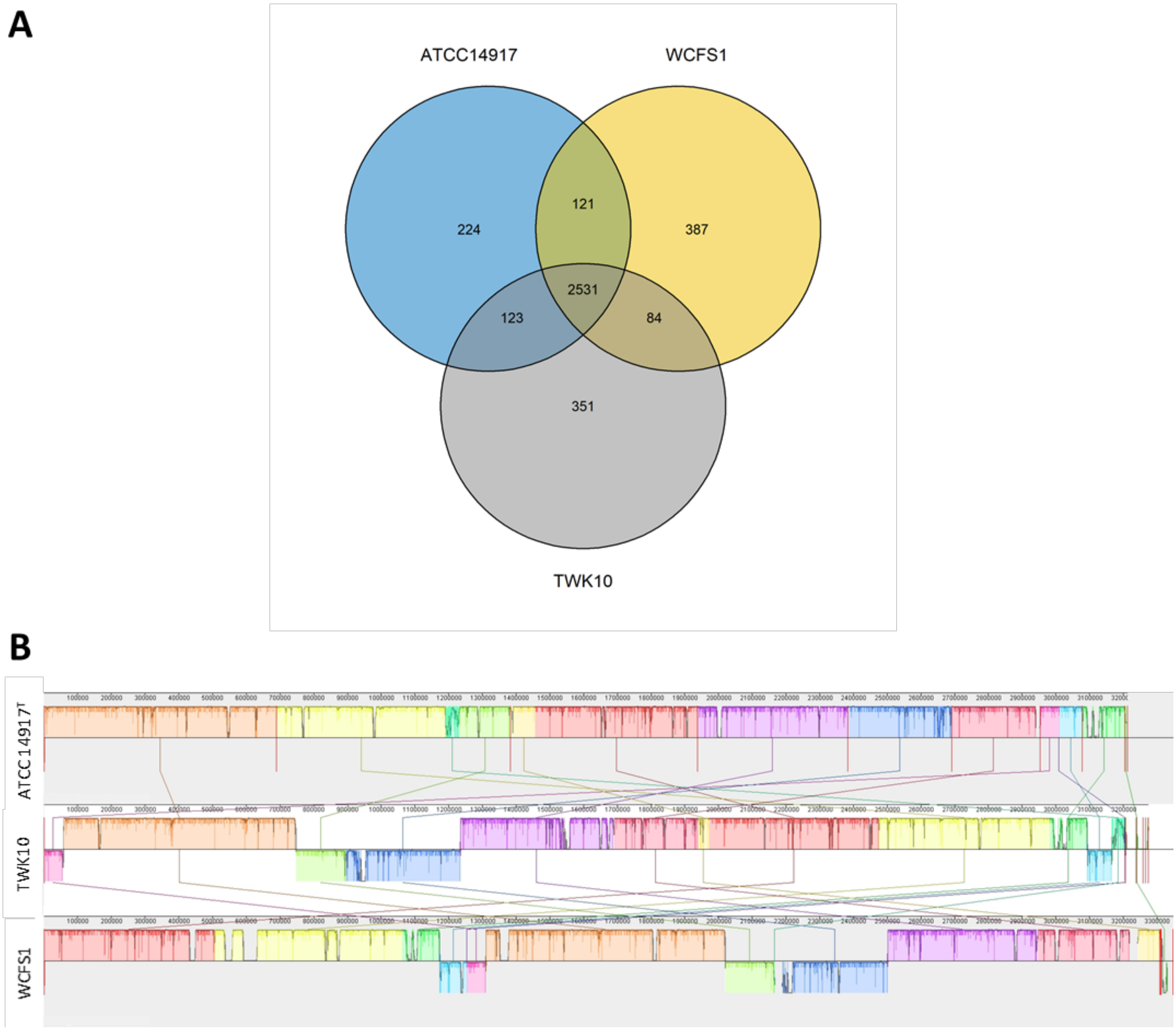

Figure S4. Genomic comparison of the TWK10 with *L. plantarum* strains, ATCC 14917^T and WCFS1. (**A**) Venn diagram representing the presence/absence of orthologous genes in the TWK10, ATCC 14917^T, and WCFS1 genomes. (**B**) Synteny of the *L. plantarum* TWK10 genome with the ATCC 14917^T and WCFS1 reference genomes obtained using MAUVE. Boxes in same color represent homologous regions (local collinear blocks; LCBs) between *L. plantarum* genomes. Homologous regions are connected by lines. Uncolored regions within the LCBs or in-between LCBs indicate the presence of strain-specific sequences.

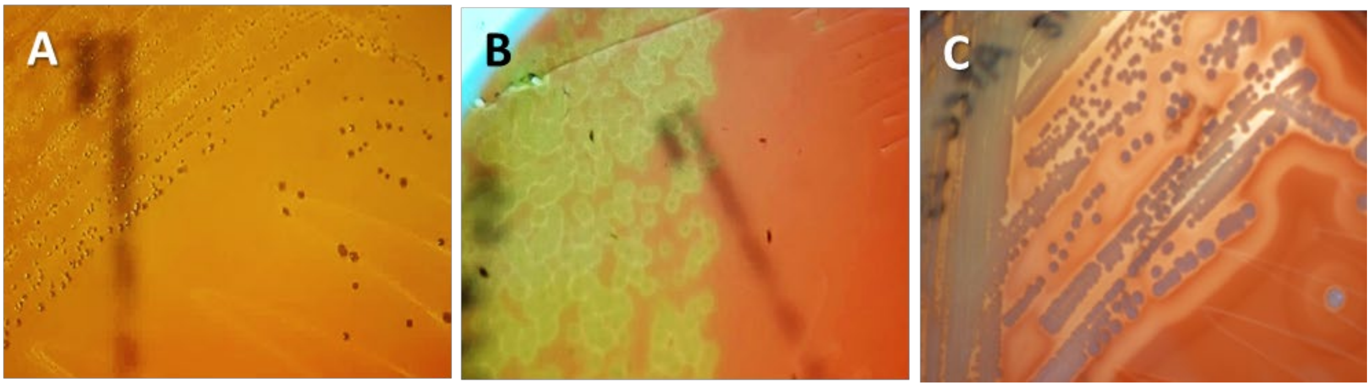

Figure S5. Hemolytic activity of TWK10. Hemolytic activity was determined on MRS agar supplemented with 5% (w/v) defibrinated sheep blood at 37 °C under aerobic conditions for 48 h. (**A**) TWK10. (**B**) α-hemolytic positive control strain, *Streptococcus pneumoniae* ATCC 6305. (**C**) β-hemolytic positive control strain, *Staphylococcus aureus* ATCC 25923.

Table S1. Average nucleotide identity (ANI) values and digital DNA–DNA hybridization (dDDH) prediction values between strain TWK10 and genetically closely related species in the genus *Lactiplantibacillus.*

	Species	Strain	Accession No.	1	2	3	4	5	6	7	8	9	10
1	*L. plantarum* subsp. *plantarum*	TWK10		**100**	99.12 *	95.55	86.10	79.89	77.49	75.06	74.26	74.89	75.23
2	*L. plantarum* subsp. *plantarum*	ATCC 14917^{T}	GCA_0001437 45.1	92.7†	**100**	95.41	85.97	79.68	77.31	74.73	74.22	74.81	75.11
3	*L. argentoratensis*	DSM 16365^{T}	GCA_0036411 65.1	62.9	62.9	**100**	85.79	80.22	77.14	74.96	74.26	74.76	75.25
4	*L. paraplantarum*	DSM 10667^{T}	GCA_0036411 45.1	31.6	31.1	31.1	**100**	80.06	77.68	75.16	74.22	74.97	75.19
5	*L. Pentosus*	DSM 20314^{T}	GCA_0036411 85.1	24.4	23.8	24.7	24.2	**100**	77.07	74.88	74.04	74.76	75.32
6	*L. herbarum*	TCF032-$E4^{T}$	GCA_0010390 45.1	22.7	22.5	21.8	22.7	22.1	**100**	74.76	74.69	74.81	75.26
7	*L. daowaiensis*	203-3^{T}	GCA_0054050 85.1	22.0	21.0	21.5	21.4	21.8	20.3	**100**	79.31	78.00	76.92
8	*L. dongliensis*	11050^{T}	GCA_0054051 05.1	21.5	21.2	21.1	21.5	21.7	20.6	22.5	**100**	76.97	75.85
9	*L. mudanjiangensis*	218-3^{T}	GCA_0054053 85.1	20.7	20.5	20.3	20.5	20.8	20.1	23.0	21.7	**100**	76.50
10	*L. fabifermentans*	DSM 21115^{T}	GCA_0004989 55.1	22.2	21.8	21.8	21.6	22.0	21.3	22.5	22.4	21.5	**100**

The values on the upper right are the *OrthoANI values (%), and the values on the lower left are the dDDH† values (%).

Table S2. Genes associated with general COG functional categories in TWK10 genome.

COG	Description	TWK10		ATCC 14917^{T}		WCFS1	
		Number of genes	%	Number of genes	%	Number of genes	%
K	Transcription	276	10.7	280	11.3	291	11.3
L	Replication, recombination and repair	240	9.3	172	7	176	6.8

G	Carbohydrate transport and metabolism	175	6.8	191	7.7	194	7.5
J	Translation, ribosomal structure and biogenesis	171	6.6	172	7	172	6.7
E	Amino acid transport and metabolism	163	6.3	161	6.5	171	6.6
M	Cell wall/membrane/envelope biogenesis	157	6.1	154	6.2	151	5.8
P	Inorganic ion transport and metabolism	122	4.7	121	4.9	123	4.8
F	Nucleotide transport and metabolism	121	4.7	122	4.9	126	4.9
C	Energy production and conversion	107	4.1	111	4.5	117	4.5
H	Coenzyme transport and metabolism	85	3.3	82	3.3	95	3.7
U	Intracellular trafficking, secretion, and vesicular transport	63	2.4	64	2.6	64	2.5
V	Defense mechanisms	63	2.4	60	2.4	63	2.4
T	Signal transduction mechanisms	58	2.2	55	2.2	53	2.1
I	Lipid transport and metabolism	50	1.9	49	2	53	2.1
O	Posttranslational modification, protein turnover, chaperones	46	1.8	47	1.9	46	1.8
D	Cell cycle control, cell division, chromosome partitioning	41	1.6	37	1.5	40	1.5
Q	Secondary metabolite biosynthesis, transport and catabolism	16	0.6	14	0.6	18	0.7
N	Cell motility	7	0.3	5	0.2	4	0.2
S	Function unknown	621	24.1	572	23.2	627	24.3
Total		2582	100	2469	100	2584	100

Table S3. Comparison of SEED subsystem features of TWK10 and *L. plantarum* reference strains, ATCC 14917^T and WCFS1.

Subsystem feature category	TWK10	ATCC 14917^T	WCFS1
Carbohydrates	230	230	248
Amino Acids and Derivatives	173	169	161
Protein Metabolism	136	118	137
Cofactors, Vitamins, Prosthetic Groups, Pigments	107	100	103
Nucleosides and Nucleotides	87	86	88
DNA Metabolism	60	51	57
Cell Wall and Capsule	57	54	77
RNA Metabolism	42	40	38
Virulence, Disease and Defense	38	38	36
Membrane Transport	34	34	34
Fatty Acids, Lipids, and Isoprenoids	34	34	36
Stress Response	21	21	20
Regulation and Cell signaling	19	16	20
Miscellaneous	14	14	14
Phages, Prophages, Transposable elements, Plasmids	10	12	10
Metabolism of Aromatic Compounds	9	5	9
Respiration	8	16	16
Phosphorus Metabolism	7	7	7
Potassium metabolism	6	5	5
Dormancy and Sporulation	6	6	6
Iron acquisition and metabolism	5	5	5
Cell Division and Cell Cycle	4	4	4
Secondary Metabolism	4	4	4
Sulfur Metabolism	3	3	3
Photosynthesis	0	0	0
Motility and Chemotaxis	0	0	0
Nitrogen Metabolism	0	0	8

Total	1114	1072	1146

Genome sequences of TWK10, ATCC 14917^{T} and WCFS1 were uploaded to the SEED Viewer server independently. Functional roles of RAST annotated genes were assigned and grouped in subsystem feature categories.

Table S4. Strain-specific SEED subsystem functions that differ between TWK10 and WCFS1.

Strain	Category	Subcategory	Subsystem	Role
TWK10	Amino Acids and Derivatives	Glutamine, glutamate, aspartate, asparagine; ammonia assimilation	Glutamine, Glutamate, Aspartate and Asparagine Biosynthesis	Asparagine synthetase [glutamine-hydrolyzing] (EC 6.3.5.4) AsnB
TWK10	Cofactors, Vitamins, Prosthetic Groups, Pigments	Riboflavin, FMN, FAD	Riboflavin, FMN and FAD metabolism	5-amino-6-(5-phosphoribosylamino) uracil reductase (EC 1.1.1.193)
TWK10	Cofactors, Vitamins, Prosthetic Groups, Pigments	Riboflavin, FMN, FAD	Riboflavin, FMN and FAD metabolism	Diaminohydroxyphosphoribosylaminopyrimidine deaminase (EC 3.5.4.26)
TWK10	DNA Metabolism	DNA repair	DNA repair, bacterial	DNA-cytosine methyltransferase (EC 2.1.1.37)
TWK10	DNA Metabolism	DNA repair	DNA repair, bacterial MutL-MutS system	MutS domain protein, family 4
TWK10	DNA Metabolism	DNA uptake, competence	DNA processing cluster	DNA topoisomerase III (EC 5.99.1.2)
TWK10	Phages, Prophages, Transposable elements, Plasmids	Phages, Prophages	Phage introns	HNH homing endonuclease
TWK10	Phages, Prophages, Transposable elements, Plasmids	Phages, Prophages	Phage packaging machinery	Phage DNA packaging
TWK10	Phages, Prophages, Transposable elements, Plasmids	Phages, Prophages	Phage tail fiber proteins	Phage tail fibers
TWK10	RNA Metabolism	no subcategory	Group II intron-associated genes	Retron-type RNA-directed DNA polymerase (EC 2.7.7.49)
TWK10	Stress Response	Osmotic stress	Choline and Betaine Uptake and Betaine Biosynthesis	Choline binding protein A
WCFS1	Carbohydrates	Central carbohydrate metabolism	Pentose phosphate pathway	Transketolase, C-terminal section (EC 2.2.1.1)
WCFS1	Carbohydrates	Central carbohydrate metabolism	Pentose phosphate pathway	Transketolase, N-terminal section (EC 2.2.1.1)
WCFS1	Carbohydrates	Monosaccharides	Deoxyribose and Deoxynucleoside Catabolism	Deoxyribonucleoside regulator DeoR (transcriptional repressor)
WCFS1	Carbohydrates	Monosaccharides	Mannose Metabolism	Alpha-1,2-mannosidase

WCF S1	Carbohydrates	Monosaccharides	Mannose Metabolism	PTS system, mannose-specific IIB component (EC 2.7.1.69)
WCF S1	Carbohydrates	Sugar alcohols	Inositol catabolism	Inosose dehydratase (EC 4.2.1.44)
WCF S1	Carbohydrates	Sugar alcohols	Inositol catabolism	Major myo-inositol transporter IolT
WCF S1	Carbohydrates	Sugar alcohols	Inositol catabolism	Myo-inositol 2-dehydrogenase (EC 1.1.1.18)
WCF S1	Carbohydrates	Sugar alcohols	Inositol catabolism	Myo-inositol 2-dehydrogenase 1 (EC 1.1.1.18)
WCF S1	Cell Wall and Capsule	Capsular and extracellular polysaccharides	Rhamnose containing glycans	Alpha-L-Rha alpha-1,3-L-rhamnosyltransferase (EC 2.4.1.-)
WCF S1	Cell Wall and Capsule	Capsular and extracellular polysaccharides	Rhamnose containing glycans	Glucose-1-phosphate thymidylyltransferase (EC 2.7.7.24)
WCF S1	Cell Wall and Capsule	Capsular and extracellular polysaccharides	Rhamnose containing glycans	dTDP-4-dehydrorhamnose 3,5-epimerase (EC 5.1.3.13)
WCF S1	Cell Wall and Capsule	Capsular and extracellular polysaccharides	Rhamnose containing glycans	dTDP-4-dehydrorhamnose reductase (EC 1.1.1.133)
WCF S1	Cell Wall and Capsule	Capsular and extracellular polysaccharides	Sialic Acid Metabolism	N-acetylneuraminate lyase (EC 4.1.3.3)
WCF S1	Cell Wall and Capsule	Capsular and extracellular polysaccharides	Sialic Acid Metabolism	Predicted sialic acid transporter
WCF S1	Clustering-based subsystems	Cofactors	Riboflavin synthesis cluster	Inner membrane protein YihY, formerly thought to be RNase BN
WCF S1	Cofactors, Vitamins, Prosthetic Groups, Pigments	Coenzyme A	Coenzyme A Biosynthesis	Aspartate 1-decarboxylase (EC 4.1.1.11)
WCF S1	Cofactors, Vitamins, Prosthetic Groups, Pigments	Folate and pterines	Molybdenum cofactor biosynthesis	Molybdenum cofactor biosynthesis protein MoaB
WCF S1	Cofactors, Vitamins, Prosthetic Groups, Pigments	Folate and pterines	Molybdenum cofactor biosynthesis	Molybdopterin-guanine dinucleotide biosynthesis protein MobA
WCF S1	Cofactors, Vitamins, Prosthetic Groups, Pigments	Folate and pterines	Molybdenum cofactor biosynthesis	Molybdopterin-guanine dinucleotide biosynthesis protein MobB
WCF S1	Cofactors, Vitamins, Prosthetic Groups, Pigments	Lipoic acid	Lipoic acid metabolism	Protein:protein lipoyl transferase
WCF S1	DNA Metabolism	no subcategory	Restriction-Modification System	Type I restriction-modification system, DNA-methyltransferase subunit M (EC 2.1.1.72)
WCF S1	DNA Metabolism	no subcategory	Restriction-Modification System	Type I restriction-modification system, restriction subunit R (EC 3.1.21.3)

WCF S1	DNA Metabolism	no subcategory	Restriction-Modification System	Type I restriction-modification system, specificity subunit S (EC 3.1.21.3)
WCF S1	Fatty Acids, Lipids, and Isoprenoids	Triacylglycerols	Triacylglycerol metabolism	Lysophospholipase (EC 3.1.1.5)
WCF S1	Nitrogen Metabolism	Denitrification	Denitrifying reductase gene clusters	Respiratory nitrate reductase alpha chain (EC 1.7.99.4)
WCF S1	Nitrogen Metabolism	Denitrification	Denitrifying reductase gene clusters	Respiratory nitrate reductase beta chain (EC 1.7.99.4)
WCF S1	Nitrogen Metabolism	Denitrification	Denitrifying reductase gene clusters	Respiratory nitrate reductase delta chain (EC 1.7.99.4)
WCF S1	Nitrogen Metabolism	Denitrification	Denitrifying reductase gene clusters	Respiratory nitrate reductase gamma chain (EC 1.7.99.4)
WCF S1	Protein Metabolism	Protein biosynthesis	Ribosome SSU bacterial	SSU ribosomal protein S14p (S29e), zinc-dependent
WCF S1	Respiration	ATP synthases	F0F1-type ATP synthase	ATP synthase F0 sector subunit b (EC 3.6.3.14)

Table S5. Bioinformatic analysis of phage sequences in the genome of TWK10.

Region	Region length (Kb)	Completeness	Total No. of Proteins	Region Position	Most Common Phage (number of gene hit)
1	45.1	intact	52	887957–933117	PHAGE_Oenoco_phiS13_NC_023560 (16)
2	33.7	intact	20	927853–961613	PHAGE_Entero_IME_EFm5_NC_028826 (3)
3	16.0	incomplete	12	1498464–1514526	PHAGE_Staphy_SPbeta_like_NC_029119 (2)
4	14.4	incomplete	10	3010218–3024667	PHAGE_Bacill_PfEFR_5_NC_031055 (1)
5	42.1	intact	55	3161904–3204038	PHAGE_Lactob_Sha1_NC_019489 (33)

Table S6. List of insertion sequences identified in the genome of TWK10.

IS	IS family	Function	Start	End
ISP2	IS1182	Transposase	2924424	2926217
ISP2			3011639	3013431
ISP2			3024616	3026410
ISP2			891008	892803
ISP2			2452980	2454772
ISP2			2576688	2578484
ISP2			1952818	1954613
ISLsa1	IS30	Transposase	2609205	2610237
ISLpl1	IS30	Transposase	1945590	1946631
ISLpl1			1835918	1836958
ISLpl1			2992253	2993294

Table S7. Hematological parameters of male and female SD rats after oral administration of TWK10 for 28 days.

	**Male**			
	**Control**	**Low dose (500 mg TWK10/kg BW)**	**Medium dose (1000 mg TWK10/kg BW)**	High dose (2000 mg TWK10/kg BW)
WBC (10^3 /μL)	13.2 ± 1.2	12.4 ± 1.4	12.0 ± 1.3	12.4 ± 1.5
RBC (10^6/μL)	9.7 ± 0.7	9.5 ± 0.4	9.5 ± 0.8	9.3 ± 0.9
Hemoglobin (g/dL)	18.8 ± 1.6	18.5 ± 0.7	18.2 ± 1.4	18.0 ± 1.6
Hematocrit (%)	54.6 ± 4.2	54.0 ± 1.9	52.5 ± 3.7	52.1 ± 4.1
MCV (fL)	56.5 ± 1.3	57.1 ± 1.6	55.4 ± 1.6	56.2 ± 1.2
MCH (pg)	19.4 ± 0.5	19.6 ± 0.5	19.2 ± 0.6	19.4 ± 0.3
MCHC (g/dL)	34.4 ± 0.5	34.3 ± 0.5	34.7 ± 0.4	34.5 ± 0.5
Platelet (10^3/μL)	1,193.1 ± 108.2	1,113.2 ± 91.5	1,132.1 ± 92.9	1,158.9 ± 90.1
Neutrophil (%)	12.1 ± 1.9	11.5 ± 1.8	13.8 ± 3.2	12.0 ± 1.7
Lymphocyte (%)	82.4 ± 2.3	83.9 ± 2.0	80.4 ± 3.7	83.3 ± 1.7
Monocyte (%)	4.3 ± 0.7	3.7 ± 0.8	4.7 ± 1.1	3.7 ± 0.6
Eosinophil (%)	1.1 ± 0.3	0.8 ± 0.3*	0.8 ± 0.4	0.9 ± 0.2
Basophil (%)	0.2 ± 0.1	0.2 ± 0.1	0.3 ± 0.3	0.2 ± 0.1
PT (sec.)	12.1 ± 1.0	12.2 ± 2.5	12.9 ± 1.9	12.3 ± 1.4
	Female			
	Control	Low dose (500 mg TWK10/kg BW)	Medium dose (1000 mg TWK10/kg BW)	High dose (2000 mg TWK10/kg BW)
WBC (10^3 /μL)	9.9 ± 1.7	11.2 ± 2.4	10.2 ± 2.1	11.8 ± 1.2
RBC (10^6/μL)	9.4 ± 0.4	9.3 ± 0.4	9.3 ± 0.4	9.4 ± 0.2
Hemoglobin (g/dL)	17.8 ± 0.7	17.4 ± 0.9	17.7 ± 0.6	18.2 ± 0.6
Hematocrit (%)	51.4 ± 2.5	50.0 ± 2.0	49.9 ± 1.9	52.2 ± 1.9
MCV (fL)	53.2 ± 1.4	53.8 ± 1.3	52.9 ± 1.5	54.6 ± 1.4
MCH (pg)	18.8 ± 0.5	18.7 ± 0.7	18.8 ± 0.7	19.2 ± 0.5
MCHC (g/dL)	35.2 ± 1.4	34.7 ± 0.7	35.5 ± 0.4	35.2 ± 0.6
Platelet (10^3/μL)	1,209.0 ± 94.9	1,155.7 ± 114.1	1,214.1 ± 175.1	1,093.8 ± 140.9
Neutrophil (%)	13.8 ± 4.0	10.8 ± 2.4	10.2 ± 3.5	12.0 ± 3.0
Lymphocyte (%)	80.9 ± 4.0	84.7 ± 2.8	85.7 ± 4.4	84.1 ± 3.5
Monocyte (%)	4.4 ± 0.8	3.5 ± 0.8	3.0 ± 1.1*	2.8 ± 0.6*
Eosinophil (%)	0.7 ± 0.2	0.8 ± 0.2	0.9 ± 0.3	0.8 ± 0.4
Basophil (%)	0.2 ± 0.1	0.2 ± 0.1	0.2 ± 0.1	0.2 ± 0.1
PT (sec.)	9.8 ± 0.4	11.0 ± 2.8	11.8 ± 6.1	9.7 ± 0.2

Data are expressed as mean ± SD, $n = 10$. Statistical significances between the values of each group and negative control were analyzed by one-way ANOVA with a post-hoc Tukey test. Results of non-parametric data, including hematocrit, neutrophil, lymphocyte, monocyte, eosinophil and basophil were analyzed by Kruskal-Wallis with a post-hoc Dunn's test. The mark (*) indicates significant difference compared with control group ($P < 0.05$). WBC, white blood cell count; RBC, red blood cell count; MCV, mean corpuscular volume; MCH, mean corpuscular hematocrit; MCHC, mean corpuscular hemoglobin concentration; PT, prothrombin time.

Table S8. Serum biochemical parameters of male and female SD rats after oral administration of TWK10 for 28 days.

		**Male**		
	**Control**	**Low dose (500 mg TWK10/kg BW)**	**Medium dose (1000 mg TWK10/kg BW)**	High dose (2000 mg TWK10/kg BW)
Glucose (mg /dL)	161.3 ± 57.0	165.7 ± 81.1	157.4 ± 53.1	144.1 ± 40.2
BUN (mg/dL)	15.8 ± 1.2	14.9 ± 1.4	14.6 ± 1.5	15.7 ± 1.1
Creatinine (mg/dL)	0.41 ± 0.03	0.41 ± 0.06	0.34 ± 0.05*	0.35 ± 0.05*
AST (U/L)	109.8 ± 17.0	132.8 ± 86.4	111.2 ± 29.5	115.9 ± 27.7
ALT (U/L)	28.9 ± 5.0	33.3 ± 19.0	28.0 ± 9.8	27.1 ± 5.7
Total protein (g/dL)	6.6 ± 0.3	6.5 ± 0.2	6.4 ± 0.3	6.5 ± 0.2
Albumin (g/dL)	4.6 ± 0.2	4.5 ± 0.2	4.4 ± 0.2*	4.6 ± 0.2
ALP (U/L)	172.5 ± 35.2	172.7 ± 25.8	172.1 ± 39.1	167.5 ± 28.9
γ-GT (U/L) [a]	< 2.0	< 2.0	< 2.0	< 2.0
Cholesterol (mg/dL)	68.3 ± 13.7	66.2 ± 11.6	68.3 ± 7.0	66.7 ± 8.1
Triglyceride (mg/dL)	59.9 ± 18.8	61.8 ± 31.4	58.7 ± 25.1	42.5 ± 10.9
Calcium (mg/dL)	12.2 ± 0.4	12.3 ± 0.7	12.1 ± 0.7	12.0 ± 0.3
Phosphorus (mg/dL)	15.2 ± 0.8	15.2 ± 1.5	14.8 ± 1.2	15.2 ± 0.9
Sodium (meg/L)	146.2 ± 1.8	146.7 ± 1.8	147.0 ± 1.8	147.3 ± 2.1
Potassium (meg/L)	8.8 ± 1.0	8.6 ± 1.2	8.0 ± 1.0	8.6 ± 0.8
Chloride (meg/L)	102.4 ± 2.1	102.8 ± 1.9	103.0 ± 1.6	104.4 ± 1.5
Globulin (g/dL)	2.0 ± 0.1	2.0 ± 0.1	2.0 ± 0.3	1.9 ± 0.1
Total bilirubin (mg/dL)	< 0.04	< 0.04	< 0.04	< 0.04
		Female		
	Control	Low dose (500 mg TWK10/kg BW)	Medium dose (1000 mg TWK10/kg BW)	High dose (2000 mg TWK10/kg BW)
Glucose (mg /dL)	116.3 ± 45.4	97.0 ± 13.8	127.3 ± 33.0	143.8 ± 45.0
BUN (mg/dL)	17.0 ± 2.3	16.0 ± 1.1	15.5 ± 1.7	16.7 ± 1.8
Creatinine (mg/dL)	0.47 ± 0.11	0.37 ± 0.08	0.33 ± 0.05*	0.39 ± 0.03*
AST (U/L)	107.8 ± 19.7	104.4 ± 16.1	92.6 ± 21.1	94.7 ± 14.4
ALT (U/L)	27.7 ± 3.7	26.1 ± 4.6	24.9 ± 5.2	22.2 ± 4.3*
Total protein (g/dL)	7.0 ± 0.3	6.8 ± 0.4	6.6 ± 0.5	6.7 ± 0.4
Albumin (m/dL)	5.2 ± 0.3	4.9 ± 0.3	4.9 ± 0.5	5.0 ± 0.4
ALP (U/L)	84.8 ± 15.5	88.0 ± 28.6	85.0 ± 14.8	91.1 ± 15.8
γ-GT (U/L) [a]	< 2.0	< 2.0	< 2.0	< 2.0
Cholesterol (mg/dL)	79.4 ± 14.4	79.7 ± 13.5	78.0 ± 13.6	84.9 ± 10.5
Triglyceride (mg/dL)	46.0 ± 20.5	34.3 ± 16.0	39.3 ± 20.8	36.2 ± 21.7
Calcium (mg/dL)	11.4 ± 0.5	11.8 ± 0.5	11.7 ± 0.6	11.8 ± 0.5
Phosphorus (mg/dL)	12.3 ± 0.5	11.7 ± 0.9	11.7 ± 1.1	11.8 ± 0.9
Sodium (meg/L)	144.8 ± 2.6	143.4 ± 2.1	142.9 ± 3.2	144.3 ± 1.2
Potassium (meg/L)	11.4 ± 1.5	11.2 ± 1.6	11.4 ± 1.7	10.4 ± 1.2
Chloride (meg/L)	99.5 ± 2.1	99.2 ± 1.4	99.5 ± 1.4	100.4 ± 2.2
Globulin (g/dL)	1.8 ± 0.1	1.9 ± 0.1	1.7 ± 0.1	1.7 ± 0.1
Total bilirubin (mg/dL)	< 0.04	< 0.04	< 0.04	< 0.04

Data are expressed as mean ± SD, *n* = 10. Statistical significances between the values of each group and negative control were analyzed by one-way ANOVA with a post-hoc Tukey test. The mark (*) indicates significant difference compared with control group ($P < 0.05$). [a] γ-GT, total bilirubin shown as lower the detection limit. BUN, blood urea nitrogen; AST, aspartate aminotransferase; ALT, alanine aminotransferase; ALP, alkaline phosphatase.
